# RNA profiles of rat olfactory epithelia: individual and age related variations

**DOI:** 10.1186/1471-2164-10-572

**Published:** 2009-12-02

**Authors:** Maud Rimbault, Stéphanie Robin, Amaury Vaysse, Francis Galibert

**Affiliations:** 1 Université de Rennes 1, Institut de Génétique et Développement de Rennes, UEB, Faculté de Médecine, Rennes, France; 2 UMR 6061 Institut de Génétique et Développement de Rennes, CNRS - Université Rennes 1, Rennes, France

## Abstract

**Background:**

Mammalian genomes contain a large number (~1000) of olfactory receptor (OR) genes, many of which (20 to 50%) are pseudogenes. OR gene transcription is not restricted to the olfactory epithelium, but is found in numerous tissues. Using microarray hybridization and RTqPCR, we analyzed the mRNA profiles of the olfactory epithelium of male and female Brown Norway rats of different origins and ages (newborn, adult and old).

**Results:**

(1) We observed very little difference between males and females and between rats from two different suppliers. (2) Different OR genes were expressed at varying levels, rather than uniformly across the four endoturbinates. (3) A large proportion of the gene transcripts (2/3 of all probes) were detected in all three age groups. Adult and older rats expressed similar numbers of OR genes, both expressing more OR genes than newborns. (4) Comparisons of whole transcriptomes or transcription profiles of expressed OR genes only showed a clear clustering of the samples as a function of age. (5) Most OR genes were expressed at lower levels at birth than in older animals, but a small number of OR genes were expressed specifically or were overexpressed in newborns.

**Conclusion:**

Not all OR genes are expressed at a detectable level. Pups expressed fewer OR genes than adult rats, and generally at a lower level; however, a small subset of OR genes were more strongly expressed in these newborn rats. The reasons for these differences are not understood. However, the specific expression of some OR genes in newborn olfactory epithelia may be related to the blindness and deafness of pups at birth, when these pups are heavily reliant on olfaction and their mother.

## Background

Olfactory receptor (OR) genes were first identified in the rat nasal epithelium by Buck and Axel in 1991 [1]. The receptors they encode play an essential role in olfaction, constituting a key initial element in a cascade of biochemical reactions that lead to odorant perception and recognition. Gene cloning and *in silico *mining of a number of mammalian genome sequences have identified about 800 OR intact genes and pseudogenes in the human genome [2,3] and up to 1500 OR genes (including pseudogenes) in the rat genome [4]. Indeed, these genes constitute by far the largest gene family in mammalian genomes.

A substantial percentage of OR genes -- 50% in humans [5], 24% in mouse [6], 20.3% in dog and 19.5% in rat [4] -- are pseudogenes. However, the distinction between pseudogenes and potentially active genes is not strictly defined: for example, a particular gene may exist as either a pseudogene or a potentially active gene, depending upon the population or the individual, as shown for dogs [7] and for humans [8].

Only a small number of OR proteins have been deorphanized, i.e. the ligand that they bind to has been identified, and the role of the vast majority of these proteins in olfaction remains undefined. Cloning experiments and microarray analyses have added an additional layer of complexity by showing that at least some pseudogenes can be transcribed [9,10] and that the transcription of OR genes is not necessarily restricted to the nasal mucosa, but is also detected in several other tissues, including testis [11] and kidney [12].

ORs are expressed on the surface of the cilia of olfactory sensory neurons (OSNs) lining the neuroepithelium in the nasal cavity, the site of odorant inhalation [13]. Each OSN expresses one OR from a single allele [14-18]; their axons extend to the olfactory bulb, where the axons of all OSN expressing the same OR converge on a single glomerulus [19-21]. The mechanism by which axons of OSNs expressing the same OR, but dispersed along the olfactory epithelium, converge on the same glomerulus is not totally understood. However, ORs that are not restricted to OSN cilia, but which are also present at the tip of the axons [22,23] contribute, together with other proteins, to the coalescence of the axons [21,24-26].

Studies based on cDNA library analysis and microarray hybridization have shown that only a subset of the mouse OR gene repertoire is expressed at detectable levels in the olfactory epithelium [9,27]. In humans, the pattern of transcription differs slightly between individuals [10]. The importance of such differences is not known. In particular, it is unclear whether these differences reflect individual differences in sensory function, possibly related to some form of anosmia or hyperosmia, or whether they reflect environmental differences. In this study, we carried out hybridization on whole rat genome microarrays, to analyze the transcriptome of the olfactory epithelium of adult Brown Norway rats of different origins and sex. The transcriptomes of these rats were then compared with those of newborn and aged rats, to investigate changes associated with aging.

## Results and Discussion

### I - The olfactory epithelium transcriptome of adult Brown Norway rats

**Figure 1:**
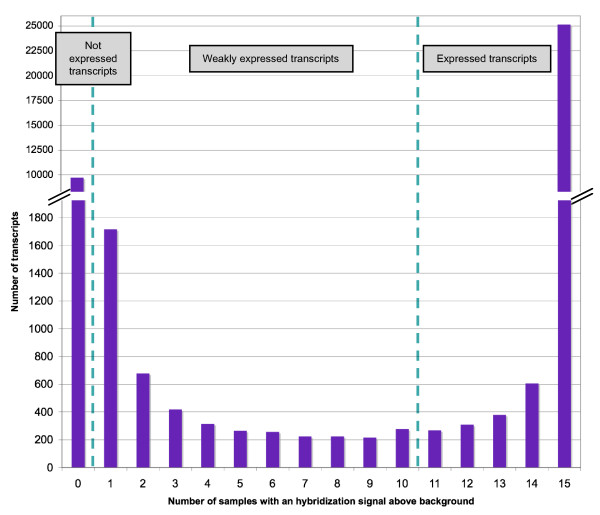
**Number of features above background**. RNA from 15 olfactory epithelia (eight rats were killed, but one olfactory RNA sample did not pass the quality control test (the RIN test) and was not further processed) were hybridized in parallel. The y-axis represents the number of transcripts plotted against the number of samples (0 to 15) with a hybridization signal above background levels, as defined by GeneSpring. Transcripts for which 11 or more of the 15 samples (73%) gave raw values above background levels were scored as "expressed". Transcripts positive in 1 to 10 samples were scored as "weakly expressed". All other transcripts were scored as "not expressed".

We determined the gene expression profiles of olfactory epithelia from six-week-old rats not exposed to a particular odorant (naive animals). We purchased four Brown Norway rats (two males and two females) from Elevage Janvier and four rats of the same strain (two males and two females) from Charles River Laboratories. After their arrival, the animals were kept in the animal house for one week and were then killed. Total RNA was extracted from left and right olfactory epithelia, labeled and used for hybridization on Agilent Whole Rat Genome 44K microarrays, as described in the Methods section. Microarrays were scanned and probes were assigned to three groups: "expressed", "weakly expressed" and "not expressed" (Figure 1).

**Figure 2:**
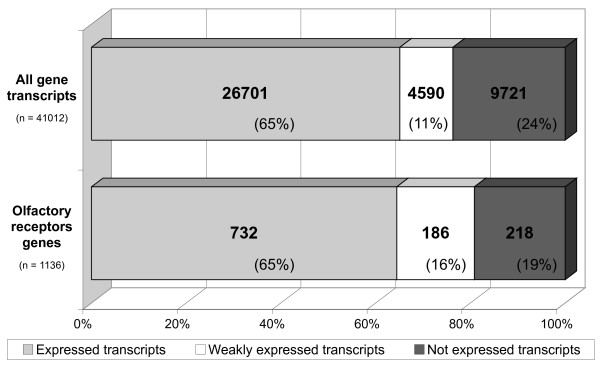
**Expression of gene transcripts and OR transcripts**. Grouping of total gene transcripts and OR transcripts into three categories: expressed, weakly expressed and not expressed. For 39,308 of the 41,012 unique probes on the array, an accession number is given in GenBank and Ensembl [45]. They correspond to 23,642 unique transcripts or genes, including 1136 OR genes. The ratios of expressed transcripts over not expressed transcripts and of expressed OR genes over not expressed OR genes are 2.75 and 3.36, respectively.

Of all the probes spotted on the array, 26,701 (65%) gave a signal above background for at least 11 of the 15 samples (≥73%) (Figure 2). These probes were considered to correspond to the set of genes expressed to detectable levels. They included 732 of the 1136 OR genes spotted on the array (from a total of 1201 rat OR genes with an intact open reading frame [4]; see additional file 1 for the OR gene list).

For probes that did not give either a positive or negative signal (above or below background levels, respectively) for all samples, we compared the signal status of each probe (positive or negative) in the right and left samples from each rat. We observed two different scenarios for the 14 samples (corresponding to seven rats from which we obtained the right and left samples): identical responses in the left and right samples, and different responses for the left and right samples. For 668 probes, we observed a 12+/2- distribution (12 samples showing positive signal and two showing negative results); for 83 of these 668 probes, the two negative results were obtained for left and right samples from the same rat. Similarly, a 12-/2+ distribution of negative and positive signal was observed for 383 probes; 105 of these probes yielded two positive results for the left and right samples from a single rat. Statistical analysis (binomial test, *p *value < 10^-4^) of the data clearly demonstrated that both negative and positive results were more frequently paired (right and left samples from the same animal giving identical results) than would be expected if the distribution were random. Probes giving different results for different samples presumably corresponded to two groups of transcripts: one corresponding to a group of genes poorly expressed at levels close to the detection limit of the method used, generating a random distribution of negative and positive results; and a second group corresponding to genes giving the same results for the right and left samples of an individual (either both positive or both negative), and thus clearly expressed by some animals and not expressed by others.

As discussed in more detail below, the set of probes corresponding to weakly expressed or not expressed genes in these experiments depends on the sensitivity of the detection method used. Nevertheless, the proportion of OR genes considered to be expressed at detectable levels in this study is consistent with previous suggestions that up to 76% of OR genes are expressed at a detectable level in the human olfactory epithelium [10].

#### • Statistical analysis (t-test)

We performed a statistical analysis (t-test, *p *value < 0.01) to identify genes that were differentially expressed between animals from two different suppliers and between males and females.

Only 10 genes, none of which encoded an OR gene, were differentially expressed between rats of the two different origins. These genes were distributed over seven different autosomal chromosomes plus the "Unknown" chromosome [28] (Table 1). Six of these 10 genes were more strongly expressed, with levels twice as high, in the Charles River rats than the Elevage Janvier rats. The other four of these genes were expressed more strongly in rats from Elevage Janvier. One gene, *Per3*, was identified twice by two independent probes, validating this finding. *Per3 *and *Dbp*, also identified among these ten genes, are both involved in circadian rhythm [29-32].

**Table 1:** Genes differentially expressed in the olfactory epithelia of rats from different suppliers

Agilent Probe	Gene Symbol	Gene Name	Genbank ID	Chromosome	RGD ID	Fold difference Charles River/Janvier
A_44_P560113	-	-	-	RNO17	-	**2.542**

A_44_P795878	-	-	-	RNO4	-	**2.271**

A_43_P16143	Per3	period homolog 3	NM_023978	RNO5	621581	**2.19**

A_44_P210809	Hiatl1	hippocampus abundant transcript-like 1	XM_225093	RNO17	1308377	**2.168**

A_44_P196198	Per3	period homolog 3	NM_023978	RNO5	621581	**2.138**

A_44_P900646	-	-	-	chrUn	-	**2.134**

A_44_P280898	Dbp	D site of albumin promoter (albumin D-box) binding protein	NM_012543	RNO1	2491	**2.094**

A_44_P739541	-	-	-	RNO14	-	**0.412**

A_44_P926835	Rab3gap2	RAB3 GTPase activating protein subunit 2	NM_001040154	RNO13	1311518	**0.4**

A_44_P240696	Nppc	natriuretic peptide precursor C	NM_053750	RNO9	620850	**0.298**

A_42_P473398	Cxcl1	chemokine (C-X-C motif) ligand 1	NM_030845	RNO14	619869	**0.237**

Genes differentially expressed in the olfactory epithelia of rats obtained from two different suppliers and ranked by fold difference. Note that *Per3 *was identified twice and that four probes correspond to non-annotated genes.

We then compared gene expression in the olfactory epithelium between males and females. Four genes, none of which encode an OR gene, were found to be expressed more strongly in female olfactory epithelium (expression levels 1.7 times higher; Table 2). Three of these genes are located on the RNOX (*Rattus Norvegicus *X) chromosome, consistent with their stronger expression in females; the fourth gene is located on RNO2. The *Eif2s3x *gene (on RNOX) was detected twice, by two independent probes spotted onto the microarrays, again validating the differential expression of this gene. Moreover, two of these four genes, *Eif2s3x *and *Utx*, have previously been reported to be overexpressed in female mouse brain [33-35]. In line with these results, studies on adult Sprague-Dawley rats [36] with Affymetrix pangenomic arrays, or on mice [27] with arrays dedicated to OR and vomeronasal (V1R only) genes have shown no detectable difference in OR gene expression between sexes.

**Table 2:** Genes differentially expressed in the olfactory epithelia of male and female rats

Agilent Probe	Gene Symbol	Gene Name	Genbank ID	Chromosome	RGD ID	Fold difference Female/Male
A_44_P1071281	-	-	AI072660	RNOX	-	**2.846**

A_44_P384683	Utx	ubiquitously transcribed tetratricopeptide repeat, X chromosome	XM_228424	RNOX	1565481	**2.082**

A_44_P379924	Ccdc39	coiled-coil domain containing 39	NM_001107667	RNO2	1306277	**1.869**

A_43_P16682	Eif2s3x	eukaryotic translation initiation factor 2, subunit 3, structural gene X-linked	XM_216704	RNOX	1561279	**1.838**

A_44_P500598	Eif2s3x	eukaryotic translation initiation factor 2, subunit 3, structural gene X-linked	XM_216704	RNOX	1561279	**1.721**

Genes differentially expressed in olfactory epithelia between male and female rats Note that *Eif2s3x *was identified twice and one probe corresponds to a non-annotated gene.

We performed hierarchical clustering and principal component analysis for the whole set of expressed gene transcripts and with OR genes only (data not shown). The absence of clear clustering using either of these two approaches, together with only a very small number of genes identified in the t-test analysis, clearly demonstrates that all animals expressed essentially the same genes to similar levels, regardless of their origin and sex.

#### • Real-time reverse-transcription PCR analysis (RTqPCR)

**Figure 3:**
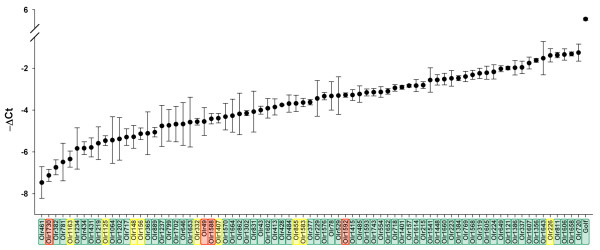
**Delta Ct values for 77 OR genes**. Results of RTqPCR (means of technical triplicate for each gene), are expressed as delta Ct values (Cycle threshold), using *Hprt *mRNA as a reference (Ct OR-Ct *Hprt*). The analyses were performed on three samples and the size of the vertical bars corresponds to the standard deviation. The curve links the mean Ct values obtained with these three samples. The name of the OR genes are indicated on the x axis: in green are genes identified as expressed, in yellow the OR genes identified as weakly expressed and in red are the OR genes identified as not expressed by microarray hybridization. As indicated by the size of the vertical bars defining the cycle threshold (Ct) values for any given analyzed RNA, the amount of mRNA differs by 0.08 to 2.2 Ct between the three samples.

Microarray hybridization represents an efficient method of comparing RNA expression levels between samples. However, as the hybridization efficiency and kinetics differ between pairs of targets and probes, it does not provide a good estimate of the relative abundance of mRNA within a sample. To overcome this limitation, we performed RTqPCR with three RNA samples to evaluate the expression level of 77 OR genes selected to represent the entire OR gene repertoire. We also included *Gαolf*, the G*α *subunit of OSN [37]. A mean difference of 6.5 Ct was observed between the most and least strongly expressed OR genes, corresponding to a 100-fold difference in transcript levels (Figure 3). A gradual change in mRNA concentration was observed between the highest and lowest amounts of transcript (Figure 3). In a previous study, a difference of up to 300-fold between the least and most strongly expressed mouse OR genes was calculated from the frequency at which OR mRNA clones were obtained [9]. We found a mean difference of 6.2 Ct between *Gαolf *and the most strongly expressed OR genes (i.e. *Gαolf *mRNA levels 80 times higher; Figure 3). *Gαolf *is expressed by all neurons, whereas a particular OR gene is expressed by only a small subset of neurons, estimated to constitute 0.1% of total OSNs [27]. Therefore, an individual neuron, particularly in the case of OSNs bearing the most strongly expressed OR, may contain more OR than *Gαolf *mRNA. However, more copies of OR than Gαolf mRNA per OSN would not be beneficial in terms of efficient signal transduction; rather, the number of Gαolf mRNA copies may differ between OSNs, with more Gαolf present in OSNs expressing larger amounts of receptor. We compared these Ct values with the microarray results. Most of the OR genes (64 out of 77), marked in green in Figure 3, were identified as expressed genes in microarray experiments. Three OR genes (marked in red) were identified as not expressed and 10 (marked in yellow) were found to be weakly expressed. A small number of the oligonucleotide pairs designed for RTqPCR experiments, corresponding to 15 additional OR genes, failed to amplify their cognate mRNA (data not shown). Eight of them were later shown to be not expressed by the microarray analyses. Altogether, these findings confirm that a majority of OR genes are indeed expressed in the olfactory epithelium, displaying considerable variation in the range of expression levels detected.

**Figure 4:**
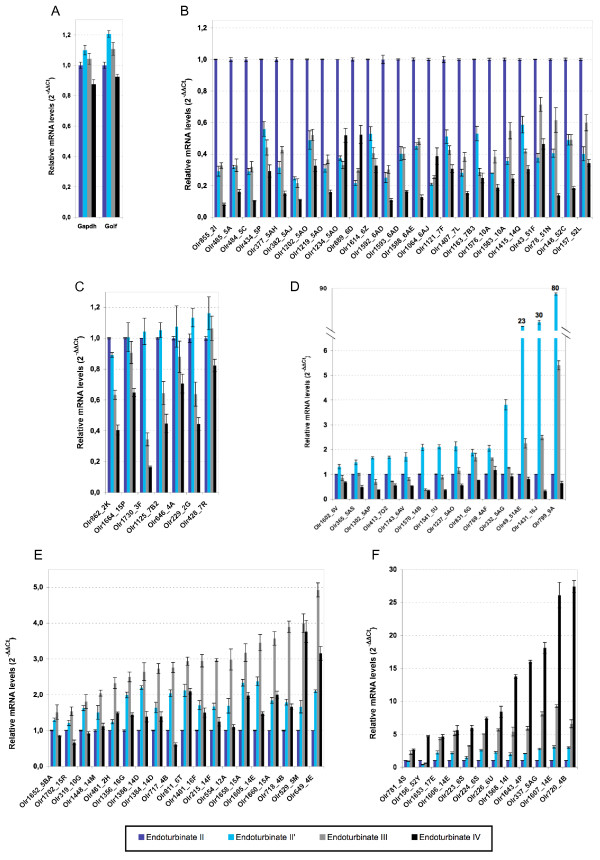
**Differential expression of OR genes in the endoturbinates**. We prepared mRNA from the four endoturbinates dissected from an adult rat. mRNA samples were used for RTqPCR (technical triplicates for each gene) for the same set of 77 OR genes. ΔΔCt values were expressed with respect to *Hprt *mRNA as internal reference and the control sample as external control as specified in [51]. Endoturbinate II', III and IV OR Ct values were normalized with respect to endoturbinate II OR Ct values, taken as a reference. The standard deviations are calculated from the triplicate values. Panel A is a control showing that *Gapdh *and *Golf *are expressed at similar levels in the four different endoturbinates. Panel B shows the Ct values for the OR genes that are more strongly expressed in endoturbinate II. Panel C shows OR genes with similar levels of expression in endoturbinates II and II'. In panel D, are shown OR genes more strongly expressed in endoturbinate II'; panel E presents OR genes more strongly expressed in endoturbinate III and, finally, panel F shows OR genes more strongly expressed in endoturbinate IV. On the x axis, OR gene family names follow gene names.

The four endoturbinates making up the olfactory epithelium were dissected individually in one adult rat and the corresponding RNA samples used for RTqPCR analysis with the same set of primers. We found that these 77 OR genes were expressed differently between these four endoturbinates, whereas *Gapdh *and *Gαolf *genes were expressed at similar levels (Figure 4). Up to 26 OR genes were more strongly expressed in endoturbinate II (Figure 4B), whereas the other ORs were more abundant in one of the other three endoturbinates (Figure 4D to 4F). We did not find a correlation between OR families and expression levels in any one endoturbinate. Previous *in situ *hybridizations with ^35^S-labeled antisense OR RNA probes have shown that neurons expressing individual receptors are topographically localized in different radial zones [19-21]. The preferential expression of a number of OR genes in a particular endoturbinate, as shown here, demonstrates another level of complexity in the olfactory epithelium structure and extends our understanding based on previous studies using electro-olfactograms [38].

### II - Changes in mRNA profile with aging

**Figure 5:**
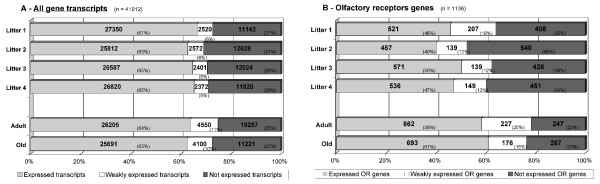
**Expression of gene transcripts and OR transcripts at various ages**. Grouping of gene transcripts and OR transcripts into three categories: expressed, weakly expressed and non expressed. In experiments describing adult olfactory epithelium mRNA profiles (Figure 1), total transcripts and OR genes were identified as being expressed when ~75% of the samples gave a corresponding hybridization signal above background levels. Similarly in the experiments described in this figure, we selected as threshold 3 in the case of each litter, 6 for the adults and 6 for the old rats. "Not expressed" total transcripts and "not expressed" OR genes did not show positive signal (above background) for any of the samples.

We investigated olfactory epithelium mRNA profiles at various ages, from birth to old age. Four female rats and their newborn pups (n = 19; 3 to 5 days old) were purchased. Newborn rats were too small for independent dissection of the right and left sides of the nasal epithelium. Therefore, RNA was extracted from one side only for each pup. RNA was also extracted from the left and right olfactory epithelium of four 22 month-old male rats kept in the animal house from the age of three weeks, and from four nine-week-old male rats. RNA samples were labeled, hybridized and the obtained data were analyzed as described above. The numbers of all transcripts and of OR genes expressed by animals of the three age groups are given in Figure 5. The distribution of expressed, weakly expressed and not expressed transcripts, when all transcripts were taken into account, did not significantly differ between rats of different ages (expressed/not expressed ratios = 2.04 to 2.45 for newborn, 2.55 for adult (or 2.74, as indicated in Figure 2) and 2.28 for old rats). However, OR gene expression profiles in newborn rats differed from the other two age groups, with "expressed OR"/"not expressed OR" ratios of 0.85 to 1.34 for the four different litters of pups, whereas it was 2.68 for adult (or 3.36 as indicated in Figure 2) and 2.59 for old rats (Mann-Whitney, *p *value = 0.057).

Comparison of the lists of gene transcripts and OR gene transcripts only showed that a vast majority of them are common to all three age groups. However, there are some notable differences between newborn and the two other groups: 332 OR genes were expressed by adults and older animals but not by newborn rats and nine OR genes were expressed by all newborn rats only (see Venn diagrams in additional file 2). Considering each litter separately, between 15 and 23 OR genes (depending upon the litter) were identified as expressed in newborns only (additional file 3).

**Figure 6:**
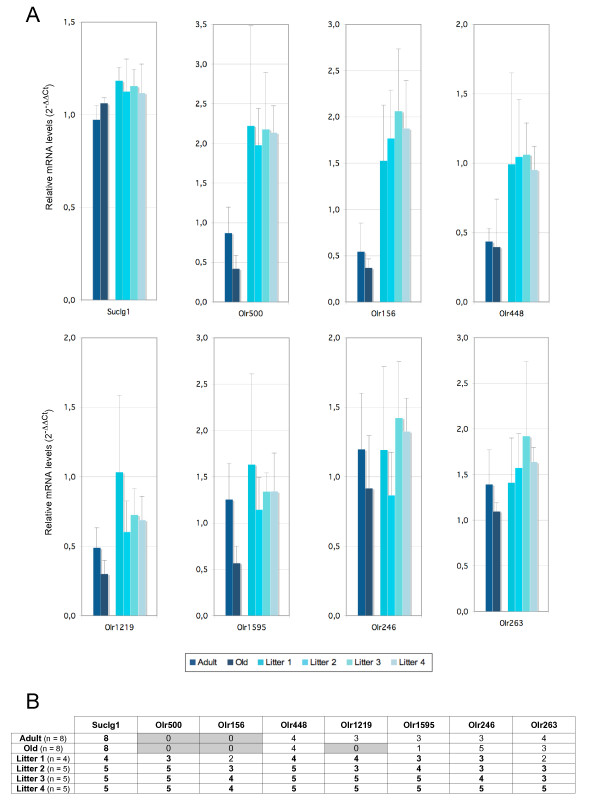
**Expression levels of OR genes expressed at birth but not in older rats**. (A) RNA samples prepared from olfactory epithelia (4 adults, 4 old rats and 19 newborns from each litter L1 to L4) were used for RTqPCR of 7 OR genes identified by microarray analysis as expressed in newborns only. Each mRNA was analyzed in triplicate and a mean value calculated. ΔCt values were calculated with respect to the *Hprt *values and ΔΔCt with respect to a control sample equated to 1. Further, the analyses were performed on four samples of the same group of age; error bars represent means ± SD (n = 4 rat RNA samples per group). (B) The table shows the number of samples giving a hybridization signal above background. For *Olr500*, taken as an example, none of the 8 adult and 8 old rat samples gave a hybridization signal above background. Conversely, all samples from the 15 newborn rats from litters L2 to L4 and three newborn rats of the four of litter L1 gave a hybridization signal above background.

We performed RTqPCR for OR genes for which expression was detected only at birth and for which we could design suitable pairs of primers (Figure 6). The results obtained from the four adult rats, four old rats and the 19 newborns displayed large standard deviations, in part due to individual variation but also due to the low level of expression typical of OR genes. Nevertheless, some of these genes tended to be more strongly expressed in pups than in adults or old rats. This effect was particularly marked for *Olr500*, *Olr156 *and *Olr448*. We then selected nine of the 28 OR genes identified as not expressed or very weakly expressed in pups (≤1 probe only - out of 19 - with hybridization levels above background) but as being expressed in adults and older rats. RTqPCR data confirmed that they all had a much lower level of expression in pups than in adults or older rats (additional file 4).

**Figure 7:**
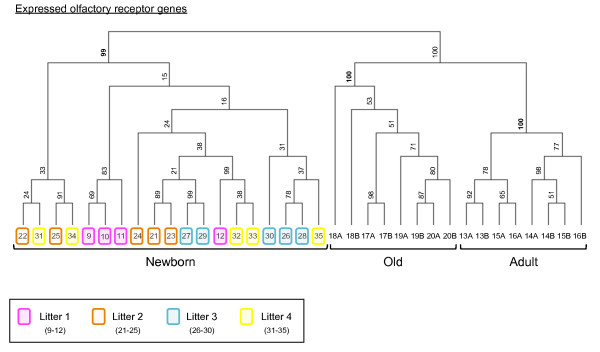
**Hierarchical clustering of samples for OR genes expressed in olfactory epithelium in the three age groups of rats**. Numbers at the nodes (range = 1 to 100) indicate support (bootstrap value) for the clustering. Note the high bootstrap values at each node corresponding to the age groups but their much lower values for the downstream branches.

The 23,780 transcripts and 393 OR genes expressed by the three age groups were subjected to hierarchical clustering (additional files 5 and 6 and figure 7). Well defined clusters were obtained for the three age groups, with high bootstrap values. Principal component analysis (PCA) analysis performed with the same data gave the same result (not shown). This complete separation of the clusters corresponding to the three age groups demonstrated clear differences in the expression levels of the genes expressed by all animals between these groups. We also observed a tendency for pups of the same litter to form clusters when all transcripts were taken into account. However, this clustering effect was far from robust (low bootstrap values) when only OR genes were considered.

**Figure 8:**
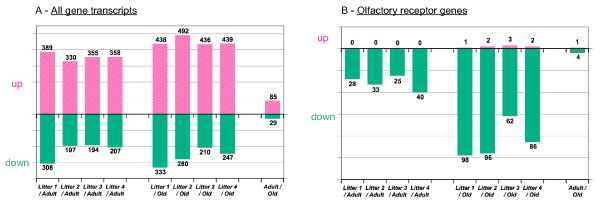
**Up- and down-regulated transcripts and OR genes**. Numbers of up- and down-regulated transcripts and OR genes (fold change ≥ 5) identified by pair-wise comparisons between age groups.

We then carried out a statistical analysis of these data (t-test, *p *value < 0.01). This showed that 2.0% of the gene transcripts were differentially expressed, with at least a five-fold difference in mRNA levels between newborns and the two other groups (adult and old animals, Figure 8). Only 0.25% of the transcripts showed differential expression between adult and old rats. Gene ontology (GO) classification analysis of the genes that were up-regulated in newborn rats showed that most of these genes were related to the "Cell Cycle" and "Neurogenesis", whereas the genes up-regulated in adults and old rats were related to the sensory perception of smell. Genes that were less strongly expressed in old rats than in adult rats were classified into GO categories associated with "Developmental processes", "Organ division" and "Ossification" (see also additional file 7 for a complete list of GO terms). Similarly, many OR genes (Figure 8) were identified as less strongly expressed in newborns than in animals of the two other groups, but eight OR genes were found to be overexpressed at birth. *Olr1079 *and *Olr1055 *were particularly strongly expressed in all newborns, with transcript levels between 4.6 and 7.7 times higher than in old rats and between 3.2 and 4.8 times higher than in adult rats (Table 3). The expression levels of another OR gene, *Olr1119*, were found to be higher in all newborns than in adults and, for three of the four litters, than in old rats (Table 3). Three OR genes (*Olr336*, *Olr921 *and *Olr581*) were overexpressed in pups from litters L2 to L4; one OR gene, *Olr441*, was overexpressed in litters L2 and L3; and another OR gene, *Olr995*, was overexpressed in litter L3 only.

**Table 3:** OR genes upregulated in newborns

Gene Symbol	Family/Subfamily	L1 vs Adult	L1 vs Old	L2 vs Adult	L2 vs Old	L3 vs Adult	L3 vs Old	L4 vs Adult	L4 vs Old	Adult vs Old
**Olr1079**	7A	3.2	**6.1**	3.9	**7.3**	4.1	**7.7**	3.6	**6.8**	-
**Olr1055**	6AO	3.7	4.6	3.8	4.7	4.8	**6.0**	4.2	**5.2**	-
**Olr1119**	7B4	3.0	-	2.9	2.9	4.4	4.4	3.9	3.9	-

**Olr336**	5AF	-	-	2.7	2.7	2.1	2.2	2.1	2.1	-
**Olr921**	6D	-	-	3.2	**5.4**	3.4	**5.8**	2.5	4.3	-
**Olr581**	5O	-	-	3.5	2.4	3.7	2.4	3.0	-	-

**Olr441**	5F	-	-	2.1	2.0	2.6	2.5	-	-	-
**Olr995**	6D	-	-	-	-	2.3	2.2	-	-	-

List of OR genes found to be up-regulated in newborn rats by t-test analysis (*p *value < 0.01). Fold differences are expressed with respect to adults and old rats. Figures in bold pointed to fold differences > 5 and "-" mean that the t-test analysis gave no significant *p *value.

**Figure 9:**
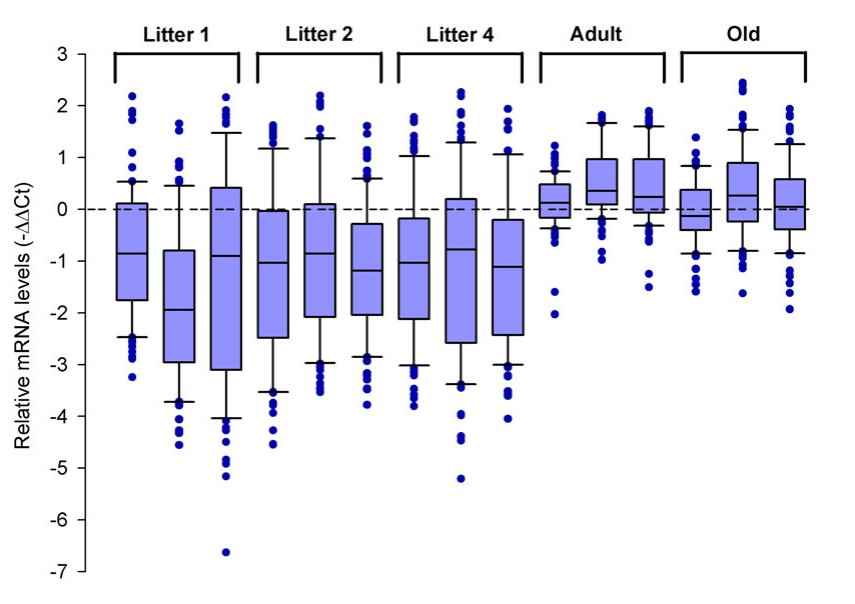
**Box plots of RTqPCR data**. The RTqPCR results detailed in Figure 10 are presented as box plots. Note the lower positions of the medians for the newborn samples and the greater dispersion of ΔΔCt values. A Mann-Whitney test performed with the 77 values calculated for the different samples confirmed that the data obtained for newborns differed from those obtained for the old and adult groups (*p *values < 10^-10^).

Next, we performed RTqPCR for the set of the 77 OR genes described above, to compare their expression levels at birth, adulthood and in old age in the olfactory epithelium RNA samples prepared from nine newborn rats from three different litters, three adult rats and three old rats. Box plots (Figure 9) showed that these 77 OR genes have a range of expression levels much greater in newborns than in older animals, and an expression level generally lower in newborns than in adult and old rats. This may be due to the immaturity of the olfactory system in young animals, consistent with the smaller number of OR genes expressed at birth.

**Figure 10:**
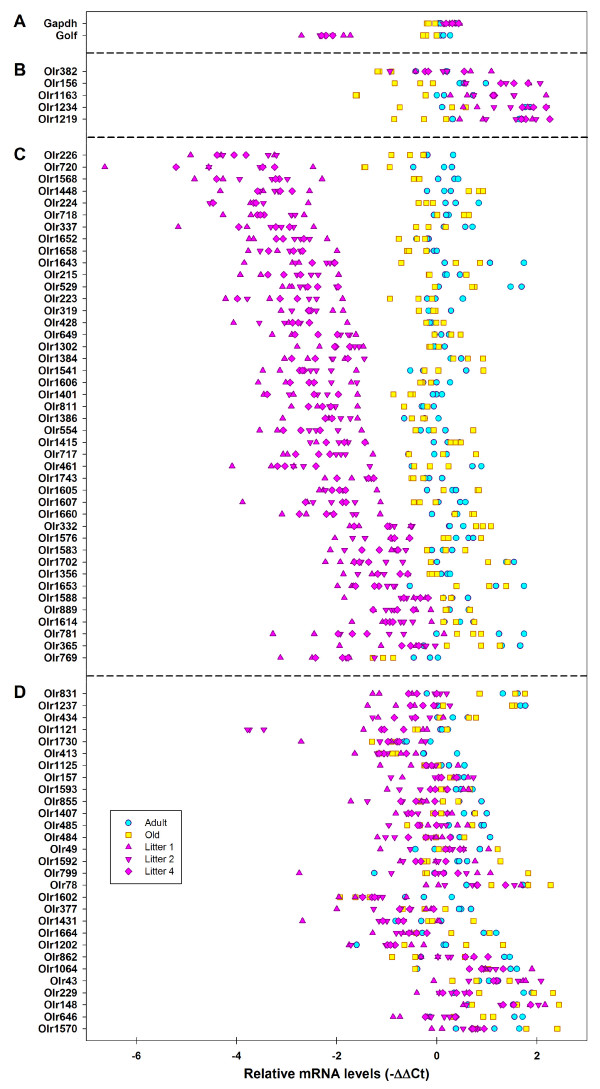
**Relative expression of a subset of 77 OR genes at three different ages**. RTqPCR was performed for 77 OR mRNAs, with olfactory epithelium mRNA prepared from 3 adult rats, 3 old rats and 9 newborn rats from three litters (L1, L2 and L4) used as a template (i.e. 15 samples, each analyzed in triplicate). Results are expressed as ΔΔCt values with *Hprt *taken as a reference (x axis). Gene names are distributed into four sectors, from top to bottom: (A) *Gαolf*, which is clearly less strongly expressed at birth and *Gapdh*, which is expressed at similar levels in all age groups, (B) 5 OR genes more strongly expressed at birth than later in life, (C) 43 OR genes far less strongly expressed at birth than at other ages and (D) 29 genes expressed to a similar extent at birth and at other ages or only slightly less strongly expressed at birth.

Of these 77 OR genes, 43 (56%) were underexpressed at birth (Figure 10C), 29 (38%) were expressed at similar levels at all ages (Figure 10D) and five (*Olr156*, *Olr382*, *Olr1163*, *Olr1219 *and *Olr1234*) were overexpressed at birth (Figure 10B). As these five OR genes were not expressed in all three age groups, they were not included in the t-test analysis. These genes should be added to the list of OR genes that are more strongly expressed at birth.

These findings -- the results of the statistical analysis (t-test; Table 3) identifying eight OR genes more strongly expressed in newborn rats than in adults, the results of RTqPCR, showing five genes overexpressed in newborn rats (Figure 10) and those presented in Figure 6 (n = 3) -- suggest that a small set of OR genes are more strongly expressed in newborn animals, with the vast majority expressed at a lower level in these animals.

## Conclusion

We report patterns of gene expression in the olfactory epithelium of adult Brown Norway rats. We found that 2/3 of the probes (i.e. 26,701 probes spotted on the arrays) gave a hybridization signal identifying genes expressed at a detectable level. The corresponding genes included 732 OR genes (65% of the total OR repertoire). We also showed by clustering analysis of the transcripts expressed in common that the pattern of expression depended on the age of the animal rather than on individual characteristics. The number of genes expressed in the olfactory epithelium, whatever their coding function, was found not to differ significantly from those reported for other tissues [39] or from the number of OR genes expressed in the mouse or human olfactory epithelium [9,10,27]. However, the classification of a gene as expressed or not expressed depends on both the detection threshold and the analytical methods used. Different arrays made with different probes may give slightly different results, as some genes not detected with one brand of microarrays may be detected by another brand due to different hybridization conditions or probe characteristics. Furthermore high-throughput sequencing, depending upon the sequencing depth, can be expected to extend the list of expressed genes [40]. Extending the list of weakly expressed OR genes is also likely to increase the ratio between the most and least strongly expressed genes.

Extending the list of poorly expressed OR genes will raise many questions. What is the minimum level at which an OR gene must be expressed to induce a signal recognized and processed by the brain? Why are some OR genes strongly expressed, whereas others are expressed only very weakly, if at all? Is this a consequence of the environment or does the panel of expressed OR genes represent the minimum required for the recognition of all relevant odorants, including those not yet encountered? In this case, under what circumstances may the transcription of weakly or not expressed OR genes be up-regulated? Answers to these questions might be obtained by subjecting rats to different olfactory environments.

Little difference was found between the olfactory epithelium mRNA profiles of individual Brown Norway adult rats of the same age, sex and origin, allowing a reference transcriptome to be defined. However, the RNA profiles of newborn, adult and old rats showed marked differences: both the lists of genes specifically expressed at each age group and the levels of expression of genes expressed in all three age groups differed between the three groups, allowing a clear clustering of the samples as a function of age. Although 22-month old rats may not be considered very old, it should be noted that the life expectancy of male Brown Norway rats is only around 31 months [41]. The gradual loss of olfactory responses in old age is probably at least in part due to the loss of central brain function [42,43]. However, the small but measurable changes in the mRNA profiles of the olfactory epithelium observed in this study between adults and old rats may also contribute to this deterioration. The smaller number of OR genes expressed and their lower levels of expression at birth would be consistent with an incomplete development of olfactory function at this age.

The Venn diagrams, t-tests and RTqPCR analyses reported here all indicate that a small number of OR genes (n = 16) were more strongly expressed or expressed exclusively in newborn rats from different litters. This number is likely to be an underestimation considering that five of the 77 OR genes taken at random were overexpressed at birth. The types of ligand they recognize are not known, but given that two- to five-day-old rats are blind and deaf, these OR genes may be important for behavior, mother-pup relationship and/or nipple recognition. These findings are consistent with the recent observation that newborn rats react to odorant exposure [44].

## Methods

### Animals

Brown Norway rats were obtained from Charles River Laboratories (L'Arbresle, France) or Elevage Janvier (Le Genest-Saint-Isle, France). Female rats with their progeny (3 to 5 days old) were purchased from Charles River Laboratories. From their arrival until the time at which they were killed, the rats were kept in the animal house (12:12 h light/dark cycles with free access to food and water) under the rules established by the Board of the Ethical Committee.

### Olfactory epithelium dissection

Rats were anesthetized with an injection of 0.3 ml/100 g body weight ketamine hydrochloride (Clorketam 1000 from Vetoquinol). They were then killed by decapitation. Rat skulls were opened through a sagittal section and right and left olfactory epithelia were quickly removed and placed separately in RA1 Buffer from the Nucleospin RNA II kit (Macherey-Nagel, Düren, Germany).

### RNA isolation

Total RNA was isolated with the Nucleospin RNA kit, according to the manufacturer's (Macherey-Nagel, Düren, Germany) instructions, which included an in-column DNase treatment before RNA elution, to ensure the absence of genomic DNA. Recovered RNA was quantified with a Nanodrop ND-1000 spectrophotometer (NanoDrop Technologies, Cambridge, UK), and RNA integrity was assessed with the RNA 6000 Nano LabChip kit, using the Agilent 2100 Bioanalyzer (Agilent Technologies, Palo Alto CA, USA). Only RNA samples with an RNA Integrity Number (RIN) greater than 8.8 were used for further analysis (RNA profiling analysis and real time reverse transcription PCR analysis). Application of this strict quality threshold resulted in the elimination of the left sample from one male adult rat from Elevage Janvier.

### Target preparation and microarray hybridization

RNA samples were labeled with the Agilent Low RNA Input Fluorescent Linear Amplification kit (p/n 5184-3523), according to the manufacturer's instructions. Briefly, 350 ng of total RNA was used as template for reverse transcription to generate cDNA, which was transcribed with T7-polymerase; cyanine-3 (Cy3)-labeled CTP was used for labeling. Cy3 labeling was monitored with a Nanodrop ND-1000 spectrophotometer and was found to be between 1.2 and 1.9 pmol/μl.

Hybridization was performed with the Agilent Gene Expression Hybridization kit (p/n 5188-5242), used according to the manufacturer's instructions. Briefly, 1650 ng of labeled cRNA from each RNA sample was mixed with Hybridization Buffer and Blocking Agent and subjected to fragmentation (by incubation for 30 min at 60°C in the dark). Hybridizations onto 4 × 44K Whole Rat Genome 60-mer oligonucleotide microarrays (G4131F) (Agilent Technologies, Palo Alto CA, USA) were performed in a rotary oven (65°C, 17 h and 10 rpm) in the dark. Slides were disassembled and washed in Gene Expression Wash Buffers I and II, according to the manufacturer's instructions, and dried with a nitrogen-filled air gun before scanning. Fifteen arrays were used for the experiment analyzing the male/female and rat origin comparisons. Thirty-five arrays were used for the "aging" comparison.

### Data acquisition and processing

Microarrays were scanned with a dynamic autofocus microarray scanner (Agilent DNA Microarray Scanner), using Agilent parameters. Feature Extraction software version 9.5 was used to extract and analyze the signals. Array results were analyzed with GeneSpring GX software version 7.3 (Agilent Technologies), via the Enhanced Agilent Feature Extraction Import Preprocessor.

Data were normalized in two steps, using the algorithms supplied with the Feature Extraction software. Data were first transformed to convert any negative value to 0.01; normalization was then performed by a per-chip 50^th ^percentile method, which normalized the data for each chip with respect to the median of the chip concerned, allowing comparison between chips. A second normalization step was applied to the results for each gene across all the arrays in the study ("normalize to median"): the median of all the values obtained for a given gene was calculated and used as the normalization standard for that gene, such that, regardless of absolute differences in the expression of the various genes, all were placed on the same scale for comparison.

The accuracy of microarray results was assessed by comparing the overall gene expression levels for each chip by box plot analysis. Each box plot was centered on zero, with comparable dynamic intensities, demonstrating the technical homogeneity of the experiment overall (data not shown).

The microarray data have been uploaded into the Gene Expression Omnibus (GEO) database (SuperSeries no. GSE15954 and samples nos. GSM400094-GSM400143).

### Expressed transcripts

Low-intensity and unreliable results were removed using a "filter on flags" feature, with standardized software algorithms classifying spots as "present," "marginal," or "absent". Spots were considered "present" only if the output was uniform, not saturated and significantly above background; spots that satisfied the main requirements but were outliers relative to the typical values for the other genes were considered "marginal". Filters were set to retain for further analysis only those values classified as "present" or "marginal".

The terms "present" or "marginal" defining the nature of the hybridization signals on each microarray should not be confused with the terms expressed transcripts, weakly expressed transcripts and not expressed transcripts defined by comparing the results obtained with the different samples, as explained in Figure 1.

### Content of the 44K Agilent microarrays

There are currently 44,012 probes on each microarrays. By annotation assignments [45], accession numbers could be assigned to 39,308 of the 39,688 probes for which the manufacturer provided chromosomal location information: rat GenBank accession numbers were assigned for 36,383 of the probes; rat Ensembl transcript identifications (IDs) were assigned for 168 other probes; and non-rat accession numbers for 2,757 probes for which no rat annotations were available. Together, these probes encompass 23,642 unique rat accession numbers and 2,270 unique non-rat accession numbers and represent 16,947 rat Unigene IDs plus 5,941 non-rat Unigene IDs (Unigene build 166). In addition to these probes, there are a number of so-called technical probes engineered by Agilent and used by GeneSpring to ascertain the quality of the data. For additional details, please consult the Agilent website [46].

Due to uncertainties regarding the names of a number of genes that are probed by many oligonucleotides on the arrays, the term "gene transcripts", used throughout this paper, designates transcripts and genes collectively identified by these probes, except for OR genes that are annotated as such. Although some gene transcripts were probed by more than one oligonucleotide, each OR gene was probed by a single oligonucleotide.

### Selection of differentially expressed genes

We performed t-test analysis with GeneSpring software (Benjamini & Hochberg correction for false discovery rate (*p *value of 0.01)) to select genes that were differentially expressed between groups.

### Hierarchical clustering

Hierarchical support trees including bootstrap analysis with replacement after 1000 iterations were constructed with TIGR Mev v 4.2 software [47]. Numbers at the nodes (range = 1 to 100) indicate the support for the clustering. The clustering pattern was generated by Pearson Correlation with average linkage clustering.

### Functional annotation

Analysis of the enrichment of expressed genes with Gene Ontology (GO) categories (i.e. GO terms with a significantly larger number of associated genes than expected for a random distribution) was performed with NIH DAVID [48,49]. Briefly, the GenBank accession numbers of the genes of interest were uploaded to the DAVID website and analysis was carried out with the *Rattus norvegicus *gene repertoire as a reference list. GO categories with significantly larger numbers of expressed genes than expected (*p *value corrected < 0.05) were selected.

### Real time reverse transcription PCR analysis (RTqPCR)

RTqPCR was performed for a number of genes, with forward (F) and reverse (R) primers designed with Primer3 software [50] (additional file 8). Primer specificity was assessed from the monophase dissociation curves. Only pairs presenting similar efficiencies (100 ± 5%) were retained (data not shown). Briefly, the High-Capacity cDNA Archive kit (Applied Biosystems, Foster City, CA, USA) was used for reverse transcription and the Power SYBR Green PCR master kit (Applied Biosystems) was used for quantitative PCR, according to Applied Biosystems gene amplification specifications (40 cycles of 15 s at 95°C and 1 min at 60°C). Gene expression was analyzed with the ABI Prism 7900HT sequence detection system, and results were handled with the associated SDS version 2.3 software (Applied Biosystems).

*Hprt *(hypoxanthine-guanine phosphoribosyltransferase) mRNA levels did not vary significantly between groups or experiments. This gene was therefore used as an internal reference for the comparison of rats of different origins and ages. The relative amounts of gene transcripts were determined by the Ct method [51]. Each PCR was carried out in triplicate. Results from different samples were compared to a "control sample" corresponding to RNA prepared from one adult rat epithelium.

## List of abbreviations used

Cy: cyanine; DNA: deoxyribonucleic acid; GEO: Gene Expression Omnibus; GO: Gene Ontology; OR: olfactory receptor; OSN: olfactory sensory neuron; PCA: principal component analysis; PCR: polymerase chain reaction; RIN: RNA integrity number; RNA: ribonucleic acid; RT: reverse transcription; SD: standard deviation

## Authors' contributions

MR carried out molecular genetic experiments, interpreted the data and drafted the manuscript. SR carried out molecular genetic experiments. AV participated in the statistical treatment of the data. FG conceived, designed, coordinated the study and helped write the manuscript. All authors read and approved the final manuscript.

## Supplementary Material

Additional file 1 **List of the 1136 OR genes that are probed in the 44K Agilent microarray**. This XLS document contains a list of the 1136 OR genes that are probed in the 44K Agilent microarray. Click here for file

Additional file 2 **Venn diagrams of transcripts and OR genes expressed in rats of the different groups of age**. This PDF document displays Venn diagrams of transcripts and OR genes expressed in rats of the different groups of age. Click here for file

Additional file 3 **Venn diagrams of OR genes expressed in newborn rats**. (A) Numbers of expressed OR genes, deduced by microarray hybridization of the RNA samples prepared from newborns of four different litters compared to the OR genes expressed by adults and old rats. (B) Names of OR genes expressed in all newborn rats but not in adults and old rats. Comparisons of the OR genes expressed in each litter with the other two age groups showed that 15 to 23 OR genes per litter were expressed exclusively in newborn rats. Interestingly, 9 of these OR genes were identified in newborn rats from all litters, four OR genes in litters L2, L3 and L4 and two OR genes in litters L1, L3 and L4. This suggests that newborn animals express a characteristic set of OR genes that is not expressed in older rats. Click here for file

Additional file 4 **mRNA levels of nine OR genes identified by microarray as expressed in adult and old animals but not in newborns**. Diagram showing RTqPCR results for nine OR genes randomly selected from the 28 OR genes not expressed at birth but expressed in adult and old animals; *Suclg1*, which was found on microarray analysis to be expressed to a similar extent in all the age groups tested, was used as a control in this experiment. Each mRNA was analyzed in triplicate and a mean value calculated. ΔCt values were calculated with respect to the *Hprt *values and ΔΔCt with respect to a control sample equated to 1. Further, the analyses were performed on four samples of the same group of age; error bars represent means ± SD (n = 4 rat RNA samples per group). Click here for file

Additional file 5 **Hierarchical clustering of samples using all transcripts for which mRNA was detected in all three groups of age**. Numbers at the nodes (range = 1 to 100) indicate support (bootstrap value) for the clustering. Click here for file

Additional file 6 **Comparison of expression level between and within age groups**. These two figures illustrate the close level of mRNA expression observed between samples prepared from animals of the same group of ages. Figure A: the microarray expression levels of three OR mRNA taken at random are compared between the 19 pup samples from 4 litters, 8 samples from 4 adults and 8 samples from 4 old rats. Figure B is a color code hierarchical clustering representation of the profiles of the 393 OR mRNA expressed in common (see additional file 2) within the 35 samples prepared from newborn, adult and old rats. Click here for file

Additional file 7 **GO terms characterizing the transcripts and OR genes up- or downregulated in the three different age groups**. Differentially expressed genes: Newborn/Old rats (up-expressed in newborn rats/foldchange > = 5) Click here for file

Additional file 8 **Oligonucleotides used for RTqPCR**. This XLS file contains Oligonucleotides used for RTqPCR. Click here for file
