# Supplementary figures and images for: RNA profiles of rat olfactory epithelia: individual and age related variations
Source: BMC Genomics. 2009 Dec 2;10:572. doi: 10.1186/1471-2164-10-572 (PMC2797534; doi:10.1186/1471-2164-10-572)

## A Expressed gene transcripts

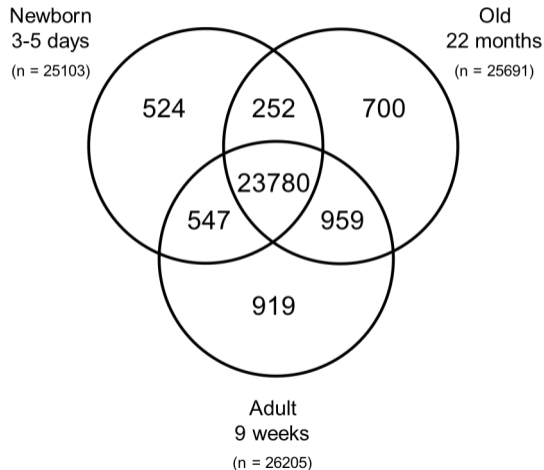

## B Expressed olfactory receptor genes

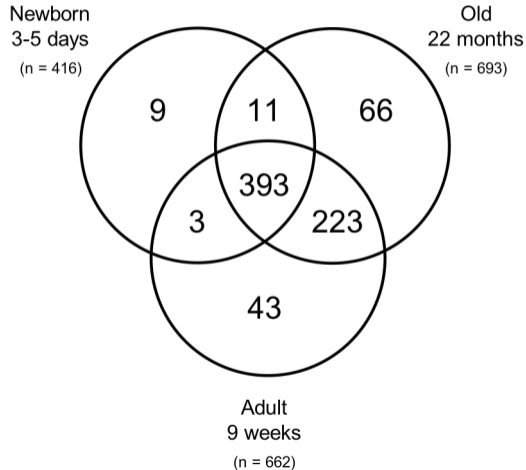

Supplement: Additional file 2 — Venn diagrams of transcripts and OR genes expressed in rats of the different groups of age. This PDF document displays Venn diagrams of transcripts and OR genes expressed in rats of the different groups of age. [file 1471-2164-10-572-S2.PDF]

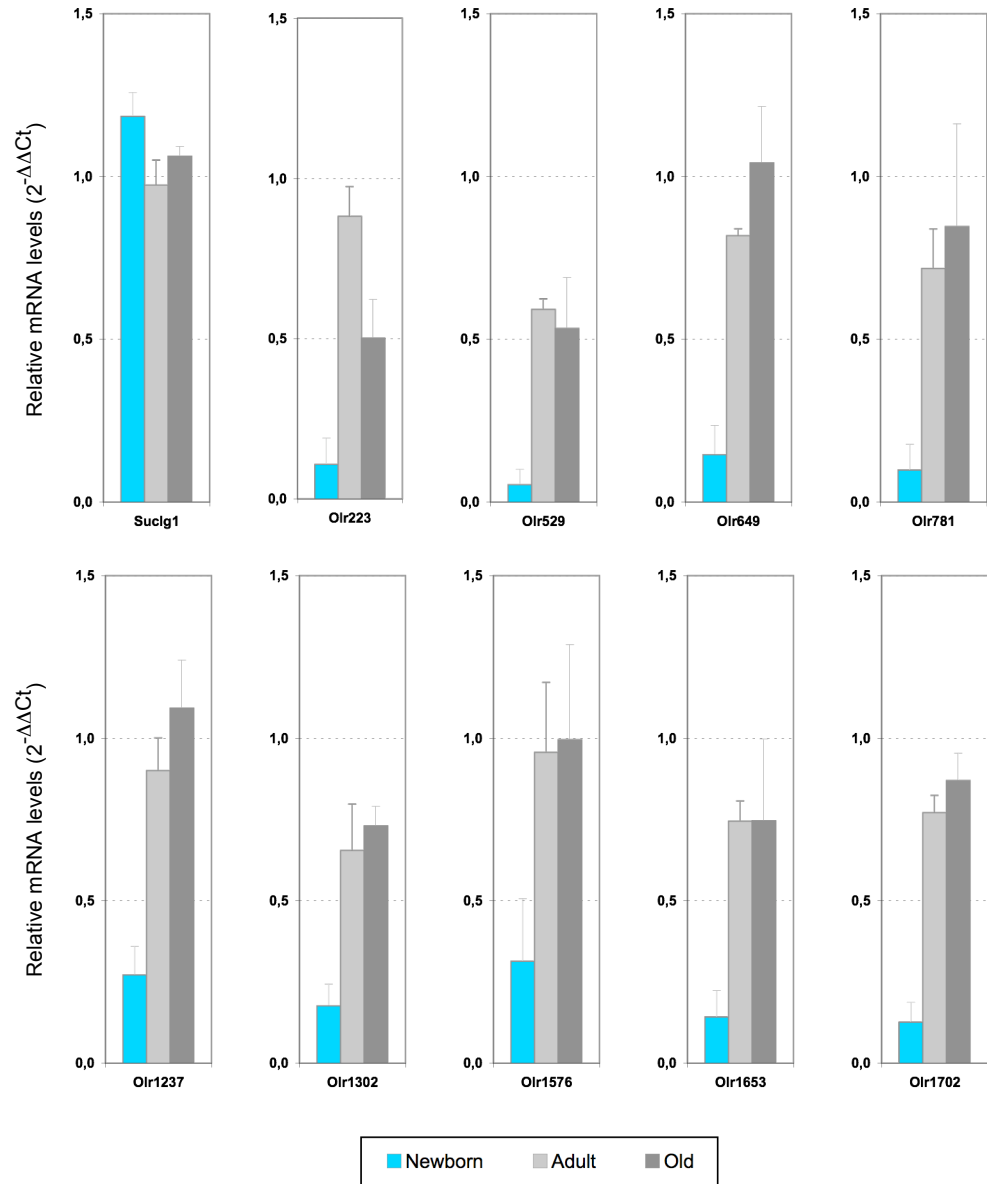

Supplement: Additional file 4 — mRNA levels of nine OR genes identified by microarray as expressed in adult and old animals but not in newborns. Diagram showing RTqPCR results for nine OR genes randomly selected from the 28 OR genes not expressed at birth but expressed in adult and old animals; Suclg1, which was found on microarray analysis to be expressed to a similar extent in all the age groups tested, was used as a control in this experiment. Each mRNA was analyzed in triplicate and a mean value calculated. ΔCt values were calculated with respect to the Hprt values and ΔΔCt with respect to a control sample equated to 1. Further, the analyses were performed on four samples of the same group of age; error bars represent means ± SD (n = 4 rat RNA samples per group). [file 1471-2164-10-572-S4.PDF]

## Expressed gene transcripts

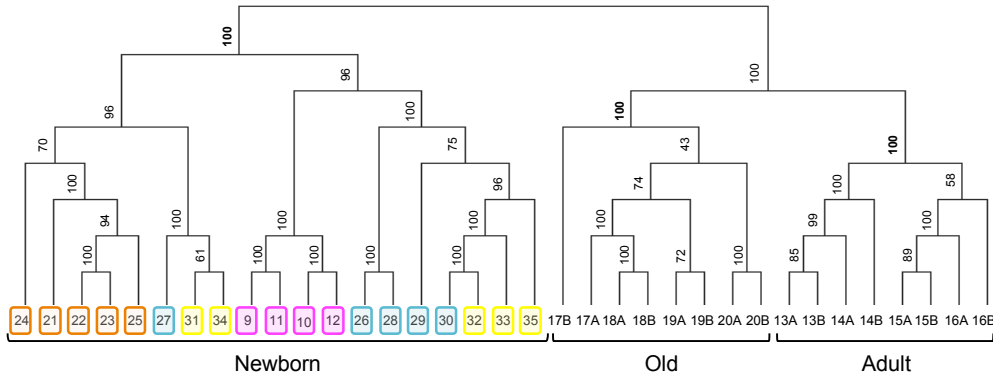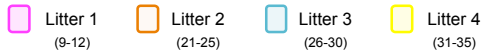

Supplement: Additional file 5 — Hierarchical clustering of samples using all transcripts for which mRNA was detected in all three groups of age. Numbers at the nodes (range = 1 to 100) indicate support (bootstrap value) for the clustering. [file 1471-2164-10-572-S5.PDF]

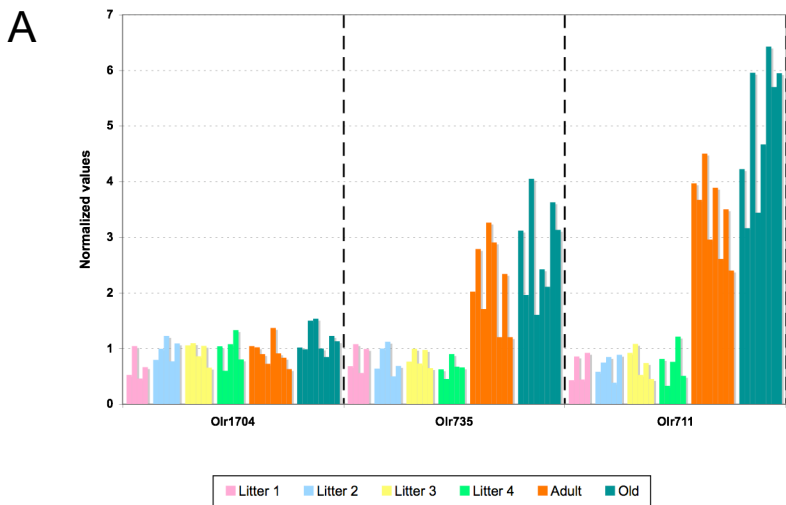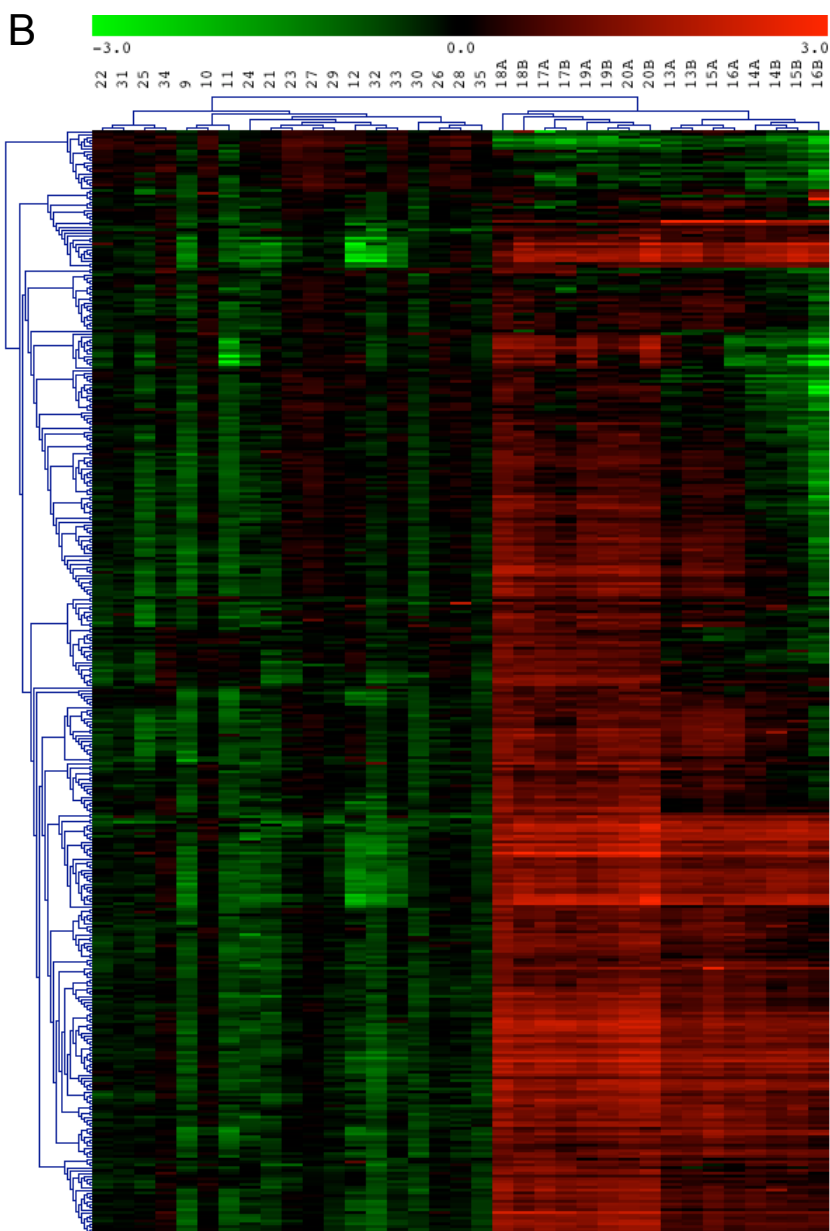

Supplement: Additional file 6 — Comparison of expression level between and within age groups. These two figures illustrate the close level of mRNA expression observed between samples prepared from animals of the same group of ages. Figure A: the microarray expression levels of three OR mRNA taken at random are compared between the 19 pup samples from 4 litters, 8 samples from 4 adults and 8 samples from 4 old rats. Figure B is a color code hierarchical clustering representation of the profiles of the 393 OR mRNA expressed in common (see additional file 2) within the 35 samples prepared from newborn, adult and old rats. [file 1471-2164-10-572-S6.PDF]
