# Supplementary material for: RNA profiles of rat olfactory epithelia: individual and age related variations
Source: BMC Genomics. 2009 Dec 2;10:572. doi: 10.1186/1471-2164-10-572 (PMC2797534; doi:10.1186/1471-2164-10-572)
Supplement: Additional file 3 — Venn diagrams of OR genes expressed in newborn rats. (A) Numbers of expressed OR genes, deduced by microarray hybridization of the RNA samples prepared from newborns of four different litters compared to the OR genes expressed by adults and old rats. (B) Names of OR genes expressed in all newborn rats but not in adults and old rats. Comparisons of the OR genes expressed in each litter with the other two age groups showed that 15 to 23 OR genes per litter were expressed exclusively in newborn rats. Interestingly, 9 of these OR genes were identified in newborn rats from all litters, four OR genes in litters L2, L3 and L4 and two OR genes in litters L1, L3 and L4. This suggests that newborn animals express a characteristic set of OR genes that is not expressed in older rats. [file 1471-2164-10-572-S3.PDF]

A

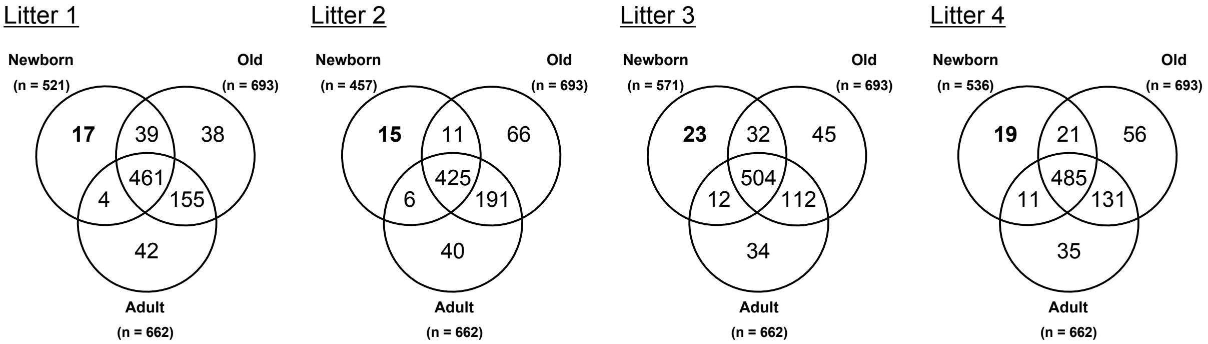


B

| Agilent Probe | Litter 1 | Litter 2 | Litter 3 | Litter 4 | Gene Symbol | Family / Subfamily | Map |
|---|---|---|---|---|---|---|---|
| A_44_P220334 | + | + | + | + | Olr1729 | - | 20p12 |
| A_44_P233712 | + | + | + | + | Olr246 | 5Y | 1q33 |
| A_44_P233726 | + | + | + | + | Olr962 | 6AK | 7q11 |
| A_44_P236036 | + | + | + | + | Olr1595 | 6AD | 13q24 |
| A_44_P293303 | + | + | + | + | *XM_345782* | 6D | chr7 |
| A_44_P463570 | + | + | + | + | Olr1219 | 5AO | 8q21 |
| A_44_P465956 | + | + | + | + | Olr922 | 6AK | 7q11 |
| A_44_P259882 | + | + | + | + | Olr448 | 5N | 3q24 |
| A_44_P198580 | + | + | + | + | Olr500 | 5A | 3q24 |
| A_44_P111786 | | + | + | + | Olr201 | 52AD | 1q33 |
| A_44_P219721 | | + | + | + | Olr263 | 5Y | 1q33 |
| A_44_P323236 | | + | + | + | Olr698 | 4B | 3q24 |
| A_44_P192041 | | + | + | + | Olr156 | 52Y | 1q32 |
| A_44_P116332 | + | | + | + | Olr1338 | 5AO | 8q22 |
| A_44_P360600 | + | | + | + | Olr803 | 2F | 4q23 |
| | +6 Olr | +2 Olr | +8 Olr | +4 Olr | | | |
